# Elites Do Not Deplete – No Effect of Prior Mental Exertion on Subsequent Shooting Performance in Elite Shooters

**DOI:** 10.3389/fpsyg.2021.668108

**Published:** 2021-06-11

**Authors:** Chris Englert, Anna Dziuba, Louis-Solal Giboin, Wanja Wolff

**Affiliations:** ^1^Department of Educational Psychology, Institute of Education, University of Bern, Bern, Switzerland; ^2^Institute of Sports and Sports Science, Karlsruhe Institute of Technology, Karlsruhe, Germany; ^3^Department of Sport Sciences, Sensorimotor Performance Lab, Human Performance Research Centre, University of Konstanz, Konstanz, Germany; ^4^Sport Psychology, Department of Sport Sciences, University of Konstanz, Konstanz, Germany

**Keywords:** self-control, self-regulation, ego depletion, fatigue, sports, mental effort

## Abstract

In order to perform at the highest level, elite shooters have to remain focused during the whole course of a tournament, which regularly lasts multiple hours. Investing self-control over extended time periods is often associated with lower levels of perceived self-control strength (i.e., the subjective estimation of how much mental effort one is capable of investing in a given task) and impaired performance in several sports-related domains. However, previous findings on the effects of prior self-control efforts on shooting performance have been mixed, as elite shooters seem to be less affected by preceding self-control demanding tasks than sub-elite athletes. Therefore, the aim of the present study was to investigate the effects of self-control on shooting performance in elite shooters. Hence, we randomly assigned elite shooters to an experimental (*n* = 12) or a control condition (*n* = 11) and asked them to perform a series of 40 shots at baseline (T1) and again after a task which either did or did not require self-control (T2). Additionally, we continuously measured the shooters’ level of perceived self-control strength. We assumed that in elite athletes, shooting accuracy as well as the perceived level of self-control strength would not be significantly affected over time from T1 to T2 in both conditions. In line with our assumptions, Bayesian linear mixed effect models revealed that shooting performance remained relatively stable in both conditions over time and the conditions also did not differ significantly in their perceived levels of self-control strength. Contrary to resource-based theories of self-control, these results speak against the idea of a limited self-control resource as previous acts of self-control did not impair subsequent shooting performance in elite athletes.

## Introduction

In professional shooting, elite athletes must perform at their highest levels during the whole course of a competition in order to be successful (e.g., Di Fronso et al., 2016). Shooting tournaments are often divided into preliminary, main, and finals, with each part lasting up to 3 h (Chen and Mordus, 2018). In order to win, athletes must consistently shoot with high accuracy as single outliers might result in a bad overall performance or even in missing the final round. Therefore, not only selectively controlling but also sustaining attention is highly relevant in shooting competitions (e.g., Ihalainen et al., 2016). Sustained attention is defined as “the ability to maintain attentional focus on relevant stimuli with repeated presentation over extended periods” (Williams and Saunders, 1997, p. 174). Research has shown that tasks that necessitate sustained attention (e.g., archery) are usually accompanied by a steady decrement in performance (e.g., Milstein et al., 2005). This decrement can also be observed in shooting (e.g., Tremayne and Barry, 2001; Kim et al., 2019). For this reason, it is crucial to understand the underlying psychological processes that influence performance (e.g., Laaksonen et al., 2018).

Volitionally controlling attention over extended periods of time requires self-control, as individuals have to inhibit themselves of paying attention to distracting stimuli and instead have to force their attention to the relevant stimuli (e.g., Pageaux and Lepers, 2018; André et al., 2019). For instance, during a shooting competition, an athlete has to shield his/her attention, ignore the crowd or internal thoughts, and focus on the task at hand in order to succeed (e.g., Abernethy et al., 2007; for an overview, see Englert, 2017, 2019). However, exerting control over the self does not always work (e.g., Head et al., 2017). Sticking to the previous example, it has been shown that basketball players are less adept at controlling their attention in high-pressure situations, leading to performance impairments (e.g., Wilson et al., 2009).

But why does something as crucial as self-control appear to fail sometimes and what determines whether or not it is applied effectively? A large body of theoretical and empirical work suggests that applying self-control is an effortful process (see, for example, Westbrook and Braver, 2015; Shenhav et al., 2017; Wolff et al., 2019; Wolff and Martarelli, 2020). Recent theorizing suggests that this sense of effort (Kurzban, 2016) serves as a signal to bias behavior away from further self-control demanding tasks (e.g., Wolff and Martarelli, 2020). Accordingly, self-control allocation can be understood as a subjective reward-based choice where an individual tries to maximize the expected value of applying self-control (Shenhav et al., 2013, 2016). This expected value of control (EVC) is computed by comparing control costs (e.g., getting fatigued while trying to stay focused on a lengthy shooting task) with the expected rewards (e.g., winning an Olympic medal in shooting) of a control-demanding action. If the costs outweigh the expected benefits, no (or not enough) self-control is applied, leading to task disengagement (or reduced performance). On the other hand, even with rising costs (e.g., due to a prior self-control task), people seem to be able to maintain a high level of performance if the task is rewarding enough for the attendant costs to not outweigh its value (Muraven and Slessareva, 2003). Indeed, a recent meta-analysis indicates that the detrimental effects of prior mental exertion are less severe when the subsequent exercise is one, the participants are used to voluntarily engage in any way (Giboin and Wolff, 2019). To illustrate, a recent study with shooters showed that self-reported self-control strength decreased over time in sub-elite athletes, whereas it did not meaningfully change in elite athletes over the course of a long series of shooting trials (Englert et al., 2020). Thus, across a series of self-control demanding shooting trials, the perceived costs of applying control were markedly lower in elite athletes. In addition, elite athletes did not display a drop in performance, whereas sub-elites’ performance substantially deteriorated over the course of the shooting task. Attesting to the crucial role of self-control for shooting performance, lower levels of perceived self-control strength (i.e., the subjective estimation of how much mental effort one is capable of investing in a given task) prior to a shooting block were robustly linked to worse subsequent shooting performance for elites and sub-elites.

Taken together, these findings indicate that over the course of a self-control demanding shooting task, elites do not perceive their self-control to wane and their performance does not deteriorate. As a limitation to these findings, Englert et al. (2020) did not experimentally manipulate prior self-control exertion with a separate task that was performed before the shooting task, but simply monitored the temporal dynamics of shooting performance and self-control over the course of the shooting task. However, such a sequential two-task paradigm approach is the established design of choice to investigate causal effects of prior self-control exertion on a subsequent self-control demanding task (e.g., Englert, 2019). Here, we address this shortcoming by investigating the role of prior mental exertion in an unrelated primary self-control demanding task on subsequent shooting performance in a sample of elite shooters. Building on recent theorizing of self-control as a reward-based choice (Kurzban et al., 2013; Shenhav et al., 2013; Wolff and Martarelli, 2020) and empirical evidence suggesting that task-specific self-control costs (i.e., the self-control demands of shooting) do not cause performance to deteriorate over the course of this task (Englert et al., 2020), we expected neither elite athletes’ perceived self-control levels nor their shooting performance to be affected by prior mental exertion.

One reason for this lack of impairments in performance and perceived self-control might be that elite athletes process self-control demands more efficiently (Wolff et al., in press) or are better at applying self-control in general (Martin et al., 2016; Wolff et al., 2019). So, a prior self-control task would not be perceived as self-control demanding and in turn would not affect subsequent shooting performance. Another explanation would be that elite athletes experience prior mental exertion in an unrelated primary task as costly. However, these costs must not necessarily carry over into the EVC calculation of the secondary task. After all, this is the task they enjoy to do (high value) and are extremely proficient at doing (low task-specific self-control costs). The first explanation would be supported if primary self-control tasks of different difficulty are not perceived to differ in the self-control demands they impose. The second explanation would be supported if the primary task creates differences in perceived self-control costs, which do not translate into altered perceptions of self-control strength (and as a consequence, altered shooting performance) in the secondary task. This second research question is tested exploratively in this paper.

The current study aims at extending Englert et al.’s (2020) findings, by investigating the causal effects of a self-control demanding task on subsequent shooting performance and perceived self-control strength.

## Materials and Methods

### Participants

A total of 23 elite shooters volunteered to participate in the present study (11 women and 12 men; *M*_age_ = 19.43, *SD*_age_ = 4.11; shooting experience: *M* = 6.03 years, *SD* = 3.69; training per week: *M* = 164.32 min, *SD* = 63.63; all participants were native German speakers; see also Table 1). Each participant was a member of the National Training Centre of Baden-Württemberg, Germany. Only the best shooters of Germany are recruited as members of the National Training Centre, delivering evidence for their high levels of expertise. Nine participants were primarily air gun shooters (10 m standard distance), and 14 participants were primarily small-caliber rifle shooters (50 m standard distance). In regard to sample size and the data analytic strategy, we followed the approach and the power simulations that had been found sufficient in Englert et al. (2020). Here, as per definition, a power analysis was performed as such: We simulated plausible data samples with different numbers of subjects according to prior assumptions regarding the effect size in the shooting scenario. On each of these samples, we fitted a model that could answer our question of interest. We then calculated the frequency of detection of group difference by the model. If the detection rate was above 80% (i.e., power of 0.8), the number of subjects was deemed adequate. Participants were asked to not consume caffeine, alcohol, or nicotine up to 24 h before the testing session and to eat a healthy meal up to 1 h before taking part in the study. The participants were informed about the basic aims of the study but were blinded with respect to the specific study hypotheses. Before beginning the assessments, each participant gave written informed consent based on APA’s ethics code. The full data set can be found at https://osf.io/7bdh6/.

**Table 1:** Descriptive statistics for both groups.

Variables	Experimental group	Control group
	*n* = 12	*n* = 11
Male sex, *N* (%)	6 (50)	6 (55)
Air gun shooters	4	5
Small-caliber rifle shooters	8	6
Age in years, *M* (*SD*)	18.25 (2.38)	20.73 (5.24)
Shooting experience in years, *M* (*SD*)	4.83 (1.67)	7.34 (4.83)
Training per week in min, *M* (*SD*)	166.36 (57.67)	162.27 (71.88)

**Table 2:** Means (*M*), standard deviations (*SD*), and internal consistencies (Cronbach’s *α*) of the German 5-Item Brief State Self-Control Capacity Scale (SMS-5; Lindner et al., 2019) for each shooting block during the first and second shooting rounds.

Shooting block	First shooting round				Second shooting round			
	Experimental group		Control group		Experimental group		Control group	
	*n* = 12		*n* = 11		*n* = 12		*n* = 11	
	*M* (*SD*)	*α*	*M* (*SD*)	*α*	*M* (*SD*)	*α*	*M* (*SD*)	*α*
Baseline	5.33 (1.26)	0.877	5.38 (1.14)	0.866	5.25 (1.22)	0.852	5.20 (0.98)	0.832
1	5.27 (1.23)	0.827	5.25 (0.95)	0.760	5.23 (1.28)	0.764	5.11 (0.94)	0.782
2	5.20 (1.39)	0.872	5.25 (0.88)	0.671	4.90 (1.80)	0.914	4.91 (1.34)	0.862
3	5.18 (1.26)	0.838	5.13 (0.93)	0.674	4.77 (1.70)	0.921	4.64 (0.98)	0.683
4	5.05 (1.48)	0.901	4.82 (1.08)	0.595	4.65 (1.74)	0.893	4.38 (0.93)	0.652

*Each item of the SMS-5 was answered on a scale from 1 (“not true”) to 7 (“very true”)*.

### Design, Procedure, and Measures

In the current study, we investigated the effects of a self-control demanding task on elite shooters’ perceived levels of self-control strength and their shooting performance over time. In order to do so, we asked elite shooters to perform a shooting task (i.e., four blocks of 10 shots each) at two times of measurement (T1 and T2) and continuously measured their levels of perceived self-control strength after 10 shots each. After T1, the participants were randomly assigned to work on a task which required high levels of self-control (experimental condition) or on a task which was less effortful (control condition).

The study was conducted in single sessions at the National Training Centre of Baden-Württemberg, Germany. First, the participants delivered written informed consent, reported demographic information (age, gender, shooting experience, and training per week), confirmed that they did not consume caffeine, alcohol, or nicotine up to 24 h before the testing session, had a healthy meal up to 1 h before taking part in the study, and performed an individual warm up session for approximately 5 min.

Then, the participants were informed that they had to perform two shooting series with a transcription task in between, starting with the first series of four shooting blocks of 10 shots each on standard regulation shooting boards (i.e., 40 shots in total; T1). On the shooting board, there were 10 concentric rings, with each ring representing a certain score (i.e., 10 points for the center of the target to 0 when the board was not hit at all). In line with the official regulations of the International Shooting Sport Federation (ISSF, 2020), the shooting boards were setup at a distance of 10 m for air gun shooters and at a distance of 50 m for small-caliber rifle shooters (i.e., the dimensions of the shooting boards were identical for gun shooters and small-caliber rifle shooters).

All instructions and questionnaires were delivered as paper-pencil versions in German. To control the possibility that the primary self-control task unintentionally affected subsequent performance *via* mechanisms that were unrelated to the incurred self-control costs, we assessed task motivation as well as positive affect and negative affect in regard to the upcoming shooting task (e.g., Englert, 2019). Task motivation was measured with the subscale Effort and Importance from the Intrinsic Motivation Inventory (IMI; Ryan, 1982; German version: Stocker et al., 2019). All five items started with the phrasing “In the following shooting task…” (sample item: “…I will do my best”) and were rated on scales ranging from one (*completely inaccurate*) to seven (*completely accurate*). For the IMI as well as for the other questionnaires included in this study, we computed overall scores by averaging each participant’s answers on the specific measure so that higher scores on the respective measure always indicated higher values of the respective variable. The IMI has been frequently adopted in sport and exercise setting and has proven to be a valid measure of motivation (e.g., Goudas et al., 2000).

Positive affect and negative affect related to the upcoming shooting task were measured using the German version of the Positive and Negative Affect Schedule (PANAS; Krohne et al., 1996). The questionnaire includes 10 items for negative affect (PANASNA; sample item: “angry”) and 10 items for positive affect (PANASPA; sample item: “interested”) which had to be answered on five-point Likert-type scales (1 – *not at all* to 5 – *very much*). The validity and reliability of the PANAS have been empirically supported in several studies (e.g., Crawford and Henry, 2004).

Finally, before starting the shooting task, shooters completed the German 5-Item Brief State Self-Control Capacity Scale (SMS-5; Lindner et al., 2019), which served as our measure of perceived self-control in the given situation. The SMS-5 is a validated short version of the State Self-Control Capacity Scale (Ciarocco et al., 2007, unpublished).^1^ The five items (“I feel drained”; “I feel calm and rational”*; “I feel lazy”; “I feel sharp and focused”*; and “I feel like my willpower is gone”; *inverted item) were answered on scales from 1 (*not true*) to 7 (*very true*) in regard to the athlete’s current state (Instruction: “Please reply spontaneously to the following statements about how you feel at the moment”). We calculated overall scores by averaging each participant’s answers, with higher scores indicating higher levels of perceived state self-control strength. The validity and reliability of the SMS-5 have been supported in previous studies (e.g., Lindner and Retelsdorf, 2020).

After filling out the SMS-5, participants performed the four shooting blocks of 10 shots each on standard regulation shooting boards and were asked to always aim for the highest score. The scores were measured *via* electronic shooting systems from the company “Meyton”^2^ consisting of the software “ShootMasterII” and the electronic scoring targets “BLACK MAGIC” using LED infrared light barriers. After each block, participants reported their perceived level of self-control strength *via* the SMS-5. In total, the SMS-5 was completed at five times during this shooting session at T1 (the internal consistencies for each time of measurement are depicted in Table 1).

After a five-minute break, participants were randomly assigned to an experimental (*n* = 12) or a control condition (*n* = 11) and transcribed a neutral text on a separate sheet of paper (for this procedure, Bertrams et al., 2010) for 6 min (as this is a typical duration in this kind of research; Giboin and Wolff, 2019). In the experimental condition, participants were asked to always omit the letters “e” and “n” while transcribing the text, whereas participants from the control condition transcribed the text conventionally. Both conditions were instructed to transcribe as many words as possible in the given time while avoiding transcription mistakes. This task has been successfully applied in numerous studies to manipulate perceived levels of self-control strength (for an overview, Englert, 2017, 2019; for two recent meta-analyses, see Giboin and Wolff, 2019; Brown et al., 2020). The number of transcribed words as well as the number of transcription errors were recorded, assuming that participants from the experimental condition would transcribe fewer words and commit more mistakes due to the more challenging instructions (see also Englert et al., 2015).

Next, participants worked on a three-item manipulation check (“How effortful did you find the transcription task?”, “How difficult did you find the transcription task?”, and “How strongly did you have to regulate your writing habits?”; Cronbach’s *α* = 0.73; Bertrams et al., 2010), which had to be answered on five-point Likert-type scales ranging from 1 (*not at all*) to 5 (*very much*), assuming that the experimental condition would experience higher self-control costs.

After that, participants immediately started their second series of four shooting blocks of 10 shots each on standard regulation shooting boards (i.e., 40 shots in total; T2). As at T1, we assessed participants’ task motivation (Stocker et al., 2019), affect (Krohne et al., 1996), and their perceived levels of self-control strength (SMS-5; Lindner et al., 2019) before executing another series of four shooting blocks of 10 shots each on standard regulation shooting boards. The SMS-5 was filled out after 10 shots each. Shooting performance was again assessed *via* the electronic shooting systems. To match the data from the two shooting rounds, each participant was assigned a unique anonymous code. For both shooting rounds, we set a time limit of 40 min, which was chosen based on the regular competition time (ISSF, 2020).

Finally, we assessed participants’ trait self-control strength with the German short version of the Self-Control Scale (SCS-K-D; Bertrams and Dickhäuser, 2009; Cronbach’s *α* = 0.82), which contains 13 items (e.g., “I am good at resisting temptations”) that had to be answered on 5-point Likert-type scales (1 = *Not at all* to 5 = *Very much*). After finishing the SCS-K-D, all participants were debriefed and thanked for their participation.

## Results

### Data Analytic Strategy

As in our previous work (Englert et al., 2020), we have estimated perceived levels of self-control strength at baseline and after each shooting block. To take into account the baseline differences in the perceived levels of self-control strength between subjects and track its change over time with a better precision, we have expressed self-control measured after shooting blocks in percentage of baseline self-control (SCpercentage).

In line with calls that have been made by fellow researchers, we refrained from using traditional ANOVA to analyze the data and to rather apply linear mixed models instead (Boisgontier and Cheval, 2016). In a nutshell, linear mixed models can be understood as linear regressions within a linear regression. They incorporate the error from clusters of non-independent data points into the total error of the statistical model. This approach has substantial advantages over more traditional statistical approaches. For example, with linear mixed models, measurements that are nested within one subject can be taken into account, unbalanced or missing data can be handled, loss of information which occurs when data are simply averaged is avoided, and the partial pooling strategy allows for better parameter estimation (Boisgontier and Cheval, 2016; Nalborczyk et al., 2019).

It is advised to fully maximize the error structure of linear mixed models to reduce type I errors (Barr et al., 2013). However, it is frequently observed that such models do not converge within a frequentist paradigm, while their Bayesian equivalents tend to converge. Thus, instead of frequentist null hypothesis significance testing (NHST), we opted to employ Bayesian analyses instead. In studies with multiple groups and/or measurements, multiple comparisons represent another issue where a Bayesian framework is better suited than traditional NHST and allows the researchers to assess whether or not an effect credibly differs from a null value (Kruschke and Liddell, 2018; Nalborczyk et al., 2019). Finally, from the perspective of communicating research findings, a Bayesian framework allows for a much more intuitive interpretation of results, as, for example, a 95% credible interval indicates that the estimate has 95% of chance of being within the interval boundaries (Kruschke and Liddell, 2018).

Data cleaning and formatting were performed with Python (3.7). Statistics were performed with R (version 3.5.3). We investigated data distribution with quantile-quantile plots using the *qqplotr* R package with confidence intervals based on an inversion of the Kolmogorov-Smirnov test (Almeida et al., 2018). For all data sets, inspection of Q-Q plots did not lead us to reject the assumption of normal distribution, and thus, all statistical models were set with a normal response distribution. We performed Bayesian statistical analysis with the R package *brms* (Bürkner, 2017, 2018) and used linear mixed models to test our hypotheses (specifics of each model are described in more detail below). For all tests, we used the default priors of the package since they are non-informative and “let the data speak” (Gelman, 2009, p. 176). For each model, we used four Markov Chain Monte Carlo with 4,000 iterations per chain (2,000 for warm-up). We checked that the models converged correctly and fit adequately the data. Unless stated otherwise, we “maximized” the error structure of each model to limit type I errors according to Barr et al. (2013). To get further information from our models, we used the build-in function *hypothesis()* from the package *brms* to calculate contrasts (see the *brms* manual). This function allows the comparison of estimates distributions by subtracting one to another.

To assess, if the experimental condition was more self-control demanding than the control condition, we compared the aggregate of the three manipulation check items (MC-M), the number of transcribed words (T-words), and transcription errors (T-errors) between groups with a simple Bayesian between group comparison. To rule out differences in trait self-control between groups, we performed another simple Bayesian between group comparison with SCS-K-D as the dependent variable. Further, we wanted to compare IMI, PANASPA, and PANASNA between groups across time. For this, we used a model with an interaction between the constant effects time and group and with random intercepts by subjects: DV ~ group × time + (1 | subject). We did not add random slopes by subject across time since we had not enough data points and models could not converge.

Then, we investigated whether shooting performance was affected by the manipulation of perceived self-control strength, and if the effect of the manipulation was exacerbated by the number of shooting blocks performed. For this, we used a linear mixed model with constant effects of group, time and blocks, and interactions between these effects. We used random intercepts by subjects and random slopes across block, time, and their interactions [shooting performance ~ group × block × time + (block × time | subject)]. Block levels were considered as numeric, while time levels were considered as factor. We used the same model to assess whether SCpercentage was affected by the manipulation of perceived self-control strength and if this effect was accentuated by the number of shooting blocks.

Results are presented as such: posterior estimate mean (posterior estimate lower and upper boundaries of the 95% credible interval). The 95% credible interval represents the area of the distribution that contains 95% of the probability distribution. Here, we consider an estimate credibly different from zero if the 95% credible interval does not contain zero.

### Preliminary Analyses

**Figure 1:**
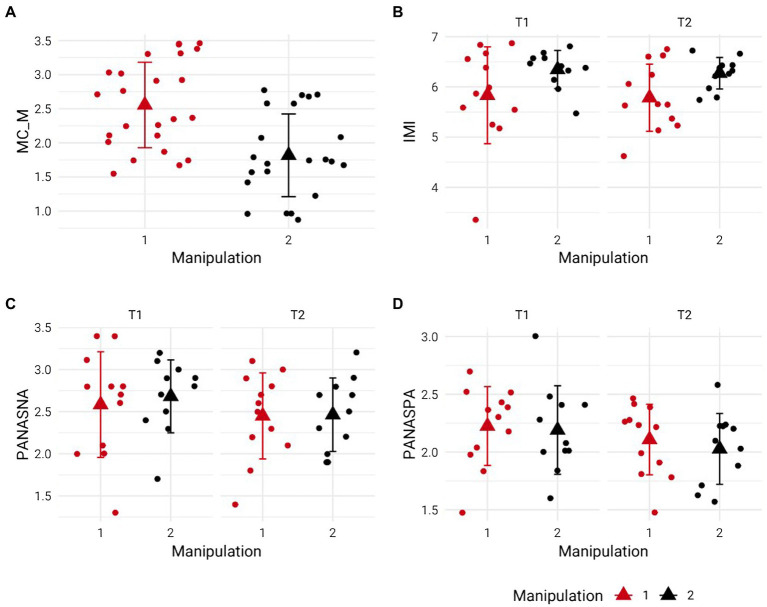
The experimental manipulation (depicted in red) was perceived as more self-control demanding than the control condition (depicted in black) **(A)** but did neither affect the motivation to complete the shooting task **(B)**, nor did it lead to changes in negative **(C)** and positive **(D)** affect. Error bars represent standard deviations.

We found that the experimental condition had a higher MC-M value (beta coefficient from experimental condition to control condition = −0.74 [−1.11, −0.37], Figure 1A), indicating that the manipulation of perceived self-control strength was effective. Further, the control group transcribed more words (beta coefficient from experimental condition to control condition = 25.85 [7.33, 44.71]) and committed less errors (beta coefficient from experimental condition to control condition = −3.95 [−6.05, −1.92]). Importantly, there were no credible group, time, and interaction effect for IMI, PANASNA, and PANASPA (Figures 1B–D, respectively, statistical results not displayed). Additionally, contrast comparisons between groups at T1 and T2 or within groups contrast comparison between T1 and T2 showed no differences. Finally, groups did not differ in regard to trait self-control, −0.10 [−0.75, 0.56].

### Main Analyses

**Figure 2:**
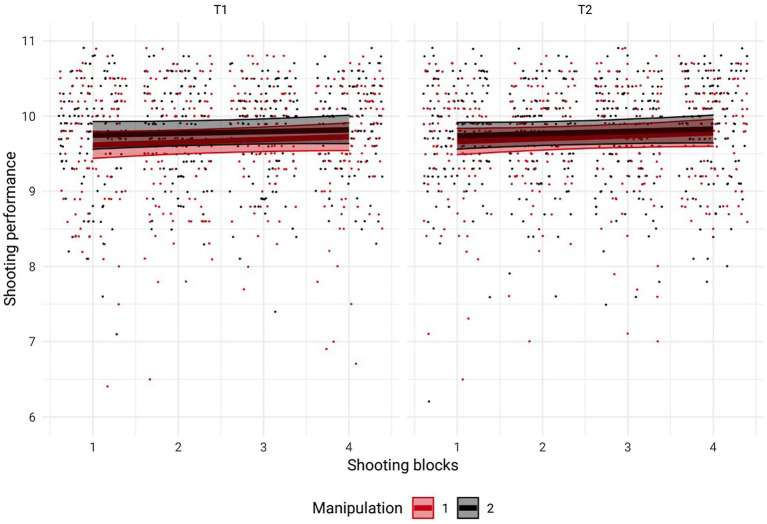
Visualization of shooting performance before (T1) and after (T2) the experimental manipulation as a function of experimental condition. Shooting performance did not change between T1 and T2 and was not affected by prior mental exertion. Data from the experimental group are depicted in red, and data from the control group are depicted in black. Error bars represent 95% credible intervals.

**Figure 3:**
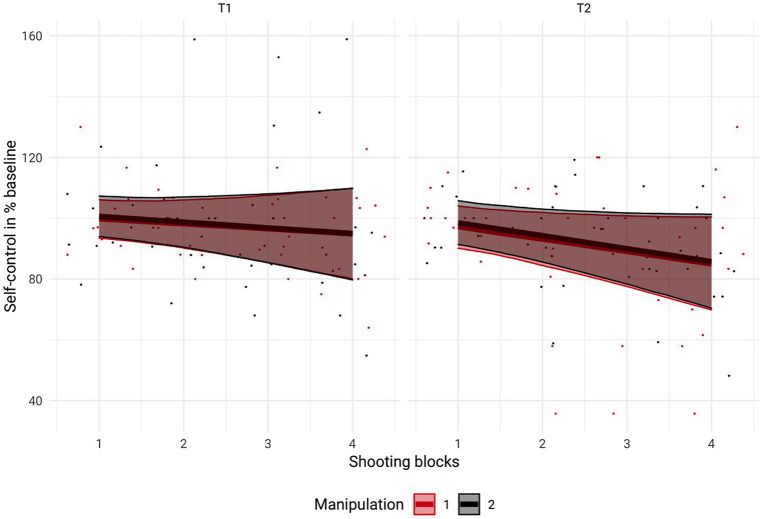
Visualization of perceived self-control strength relative to perceived self-control strength at baseline before (T1) and after (T2) the experimental manipulation as a function of experimental condition. Perceived self-control strength was not affected by prior mental exertion. Data from the experimental group are depicted in red, and data from the control group are depicted in black. Error bars represent 95% credible intervals.

As displayed in Figure 2, there was no effect of the manipulation of perceived self-control strength or shooting blocks and no interaction between these factors that affected shooting performance see also Table 2. Similarly, SCpercentage was not affected by the manipulation of perceived self-control strength, number of shooting blocks, or an interaction between these factors (Figure 3).

## Discussion

Investing high levels of self-control over extended periods of time can lead to lower levels of perceived self-control strength (e.g., Englert et al., 2020). During shooting tournaments, athletes need to remain focused over several hours suggesting that perceived self-control strength seems to play a pivotal role for top-level performance (e.g., Di Fronso et al., 2016; Chen and Mordus, 2018). A recent correlational study by Englert et al. (2020) revealed that the level of perceived self-control strength significantly decreased over the course of two one-hour shooting tasks. These decreases in perceived self-control strength were significantly related to actual shooting performance, meaning that the athletes performed worse the lower their levels of perceived self-control strength were. Interestingly, these significant drops in shooting performance and perceived self-control strength were only found in sub-elite shooters. In the present study, we built on these correlational findings reported by Englert et al. (2020) and conducted an experiment in order to dig deeper into the causal effects of a self-control demanding task on performance and the level of perceived self-control strength in elite shooters. As expected, we did not find any empirical evidence for a negative carry-over effect of the self-control demanding task on performance or the level of perceived self-control strength. How can these pattern of results be explained and how might they shed light on the mechanisms of how self-control exerts its influence?

According to one of the most prominent resource-based self-control theories – the strength model (e.g., Baumeister et al., 2007) – an individual’s self-control resources are limited: After an initial task which required high levels of self-control, one’s self-control resources should diminish over time, ultimately leading to a state of ego depletion, during which subsequent self-control demanding tasks are executed less efficiently (Englert, 2017, 2019). The current findings do not fit the assumption of a limited self-control resource, as we adopted a reliable task designed to manipulate perceived self-control strength, but shooting performance and the level of perceived self-control strength did not differ between the control and the experimental condition (Bertrams et al., 2010). Our study adds to the results of recent studies which did not find any reliable evidence for this ego depletion effect (e.g., Hagger et al., 2016). We would like to tackle four potential reasons why elite shooters did not suffer from the foregoing self-control demanding task.

First, on a methodological level, one might argue that the transcription task we adopted in our study is not an appropriate task to manipulate perceived self-control strength (Bertrams et al., 2010), as previous studies have applied other tasks with longer durations (e.g., see also Van Cutsem et al., 2017). However, we would like to point out that participants in the experimental condition did actually judge the transcription task as being more difficult, more self-control demanding, and more effortful than the control condition, indicating that the task was indeed suited to manipulate perceived levels of self-control strength (see also Englert et al., 2015). Despite the self-control demanding features of the transcription task, the elite shooters were able to remain focused in the subsequent shooting task and did not feel mentally exhausted. Nonetheless, future studies might want to apply alternative self-control demanding tasks to manipulate self-control strength, in order to replicate and extend our findings. However, this endeavor is not as easy as it might seem at first, as there are several flaws regarding the most frequently applied mentally demanding tasks (e.g., Englert et al., 2019). It is also important to mention that thus far there is no general agreement among researchers how long a self-control demanding task should ideally last, to reliably manipulate the level of perceived self-control strength (e.g., Giboin and Wolff, 2019; Englert and Bertrams, 2021; Wolff et al., 2021a). The validity of the most popular mentally fatiguing tasks should therefore be rigorously tested in future studies (e.g., Dang, 2018). In this context, we would also like to point out that future studies might want to adopt repeated measures designs when investigating the effects of effort on performance, in order to reduce between-subject variability (Charness et al., 2012). However, in the current study, it was not possible to analyze shooting performance at multiple times of measurement given the limited training time of the elite athletes.

Second, in line with recent reward-based conceptualizations of self-control, elite athletes might simply be able to perform the shooting task with less self-control costs (for a discussion, please see Wolff et al., in press). One mechanism by which task execution can become less costly is by a higher degree of automatization of the task-specific processing demands. Interestingly, this hypothesis can be supported by many neurophysiological studies indicating task- or training-specific neural adaptations following motor training (Karni et al., 1998; Doyon et al., 2002; Giboin et al., 2019b). Therefore, elite athletes are likely to have motor commands that are extremely optimized and specific for their highly trained tasks (i.e., shooting). Such specific optimizations of the motor command could be one explanation for why elite athletes incur less self-control costs for tasks that are extremely self-control demanding for non-elite athletes. Importantly, this relationship between motor costs of a physical task and the self-control costs it produces does not only apply to elite athletes. Recent psychoneurophysiological evidence shows that more efficient movement execution can improve performance in a self-control demanding physical task while being performed with less activity in brain areas that are relevant for self-control (Giboin et al., 2019a). In terms of optimizing the EVC, a reduction in task-specific control costs could skew the EVC toward applying enough control for performance to not deteriorate, although one had already applied effort toward an unrelated previous self-control task. This is in line with recent meta-analytic evidence that performance drops after self-control application are smaller if the subjects have experience in engaging in the subsequent physical self-control task (Giboin and Wolff, 2019). In regard to our second research question, this indicates that elite athletes are not immune to self-control demands in general (as evidenced by the between group difference in perceived self-control strength after the transcription task), but they seem to be able to efficiently perform the task they are experts in (i.e., shooting) regardless of prior self-control costs.

Third, a recent meta-analysis (Giboin and Wolff, 2019) showed that detrimental effects of prior mental exertion were smaller when the subsequent sporting task had a high person-situation fit (i.e., when participants were asked to do a sporting task they were proficient in as opposed to doing a task they did not regularly engage in). This effect could be even more pronounced in our sample of elite athletes, where the person-situation fit was particularly large (elites doing something they are elite at). In addition, the meta-analysis by Brown et al. (2020) showed that effects of prior mental exertion depended on the type of subsequent physical task. Observing, for example, that performance was not reliably impaired in tasks that required maximum power, whereas in other tasks that supposedly hinged more on self-control performance, was more robustly impaired. In this vein, it is possible that for highly trained shooters the sporting task did not hinge sufficiently on self-control to be robustly impaired by the prior mental exertion.

Lastly, recent work points toward a strong link between boredom and self-control (Wolff et al., 2021b), suggesting that task-induced boredom might act as a confounding self-control demand (Wolff et al., 2021c). Indeed, there is preliminary evidence showing that tasks that are designed to be less self-control demanding might be perceived as being boring (compared to tasks that are designed to be self-control demanding) and that task-induced boredom affects performance in self-control demanding tasks (Bieleke et al., 2021). Thus, another explanation as to why we observed no detrimental effects on shooting performance might be due to systematic differences in how boring the control condition was observed compared to the experimental condition. This last explanation certainly warrants further dedicated research.

Finally, we would like to offer suggestions on how to improve attention regulation and shooting performance. As mentioned in the previous paragraph, reducing the task-specific control costs could skew the EVC toward applying enough control for performance to not deteriorate. For instance, implementation intentions should help to decrease the task-specific control costs: Implementation intentions are predefined action plans which are automatically triggered if a certain situation occurs, meaning that less effort needs to be invested to execute the respective behavior (e.g., Sheeran et al., 2005). Future studies should focus on how implementation intentions can counteract the potential carry-over effects of low levels of perceived self-control strength.

In a similar fashion, the strength model argues that regular self-control training should improve self-control performance in the long run. Bray et al. (2015) asked participants to perform a tiring maximal graded cycling task at two times of measurement separated by 2 weeks. During these 2 weeks, the experimental condition had to regularly squeeze a handgrip multiple times a day (i.e., a self-control demanding task), while the control condition did not receive any additional instructions. After the two-week period, cycling performance in the experimental condition significantly improved, while participants’ performance in the control condition did not change significantly.

Taken together, the current study is in line with the correlational findings reported by Englert et al. (2020), as elite shooters seem to be less affected by a previous self-control demanding task. Future studies should continue to dig deeper into the exact mechanisms how expertise exerts its effects on self-control.

## Data Availability Statement

The data sets presented in this study can be found in online repositories. The names of the repository/repositories and accession number(s) can be found at https://osf.io/7bdh6/.

## Ethics Statement

Ethical approval was not provided for this study on human participants because ethical review and approval was not required for the study on human participants in accordance with the local legislation and institutional requirements. The patients/participants provided their written informed consent to participate in this study.

## Author Contributions

CE and AD equally contributed to the conceptualization of the study and review of relevant related work. CE, WW, and LS-G analyzed and interpreted the data. CE and WW prepared the draft manuscript, while AD and L-SG provided the critical revisions. All authors approved the final version of the manuscript and agreed with the order of presentation of the authors.

### Conflict of Interest

The authors declare that the research was conducted in the absence of any commercial or financial relationships that could be construed as a potential conflict of interest.

## Footnotes

^1^Ciarocco, N. J., Twenge, J. M., Muraven, M., & Tice, D. M. (2007). *Measuring State Self-Control: Reliability, Validity, and Correlations with Physical and Psychological Stress*. Unpublished manuscript. NJ, USA: Monmouth University.

^2^https://www.meyton.info
